# Risk Factors for Post-stroke Depression: A Meta-analysis

**DOI:** 10.3389/fnagi.2017.00218

**Published:** 2017-07-11

**Authors:** Yu Shi, Dongdong Yang, Yanyan Zeng, Wen Wu

**Affiliations:** ^1^Department of Rehabilitation, Zhujiang Hospital, Southern Medical University Guangzhou, China; ^2^Department of Neurology, Zhengzhou People’s Hospital Zhengzhou, China

**Keywords:** post-stroke depression, PSD, risk factor, systematic review, meta-analysis

## Abstract

**Background:** Stroke not only impacts patients physically but also economically. Post-stroke depression (PSD), as a common complication of stroke, always obstructs the process of stroke rehabilitation. Accordingly, defining the risk factors associated with PSD has extraordinary importance. Although there have been many studies investigating the risk factors for PSD, the results are inconsistent.

**Objectives:** The objectives of this study were to identify the risk factors for PSD by evidence-based medicine.

**Data sources:** A systematic and comprehensive database search was performed of PubMed, Medline, CENTRAL, EMBASE.com, the Cochrane library and Web of Science for Literature, covering publications from January 1, 1998 to November 19, 2016.

**Study Selection:** Studies on risk factors for PSD were identified, according to inclusion and exclusion criteria. The risk of bias tool, described in the Cochrane Handbook version 5.1.0, was used to assess the quality of each study. Meta-analysis was performed using RevMan 5.3 software.

**Results:** Thirty-six studies were included for review. A history of mental illness was the highest ranking modifiable risk factor; other risk factors for PSD were female gender, age (<70 years), neuroticism, family history, severity of stroke, and level of handicap. Social support was a protective factor for PSD.

**Conclusion:** There are many factors that have effects on PSD. The severity of stroke is an important factor in the occurrence of PSD. Mental history is a possible predictor of PSD. Prevention of PSD requires social and family participation.

## Introduction

Stroke, which has been ranked the third most deadly disease, is prevalent worldwide (Mozaffarian et al., 2016). According to the report of World Health Organization (WHO), there are 15 million people suffering from stroke every year (European Stroke Initiative Executive EUSI Writing Committee Olsen et al., 2003).

Post-stroke depression (PSD) is considered the most frequent and important neuropsychiatric consequence of a stroke that negatively affects the outcome in stroke patients. A recent systematic review indicated that the frequency of PSD is 33% (95% confidence interval [CI], 29 to 36%) (Hackett and Pickles, 2014). The Diagnostic and Statistical Manual (DSM) IV categorizes PSD as a “mood disorder due to a general medical condition (i.e., stroke)” with the specifiers of depressive features, major depressive-like episodes, manic features, or mixed features (American Psychiatric Association, 2000). Persistent depression not only increases the disease deterioration, but also causes social function defects, and increases the risk of suicide. Moreover this condition can have an adverse effect on cognitive function, functional recovery and survival. Therefore, it is urgent to study the risk factors for PSD.

In recent decades, overwhelming evidence has indicated that PSD is associated with well-known risk factors such as medical history (i.e., predisposing illness and smoking) (Shi et al., 2015), a history of mental disorders (de Man-van Ginkel et al., 2013), and stroke characteristics (Jiang et al., 2014). PSD is highly prevalent among both men and women; however, it appears that PSD is more common in women than men. Although risk factors for PSD are increasingly reported, it still lacks of evidence based on medicine. Some studies have shown the risk factors for PSD are diverse at different time periods after the stroke. For example, Robinson et al. (1986) suggested that stroke patients with left hemisphere lesions are more likely to be depressed than those with right hemisphere and brainstem lesions, which is supported by most scholars. But Sun et al. (2014) suggested that there was a correlation between depression and right hemisphere lesion 6 months after stroke. In summary, the risk factors for PSD at different time points are complex and diverse, requiring further analysis to draw a convincing conclusion.

We undertook a systematic review of studies relevant to present practice to clearly identify important risk factors for PSD. If some of these risk factors can be avoided or prevented, it would be most helpful to take preventive measures in order to promote early diagnosis, implement early and adequate treatment, and improve quality of life. To obtain a more comprehensive estimate of the putative influence of PSD, we conducted a meta-analysis to examine the risk factors for PSD associated with different time periods after the stroke.

## Materials and Methods

### Search Strategy

A systematic and comprehensive database search was performed of PubMed, Medline, CENTRAL, EMBASE.com, Cochrane library and Web of Science for Literature published from January 1, 1998 to November 19, 2016. Only papers in English and human studies were considered. We used the following terms for the search strategy: two search themes were combined using the Boolean operator “and.” The first theme, depression, combined exploded versions of the Medical Subject Headings (MeSH) depression, depressive disorder, or depressive disorder, major. The second theme, stroke, combined exploded versions of the MeSH terms stroke, cerebrovascular disorders, or intracranial embolism and thrombosis.

### Eligibility Criteria

Eligibility criteria accorded with the PICOS (participants, interventions, controls, outcomes, and studies) framework, as follows:

#### Participants

Participants were non-hospitalized adults with no history of stroke or transient ischemic attack (TIA) at the time of study initiation.

#### Interventions

The intervention variable was defined as depression, whereas the assessment of depression had to be prospectively performed at baseline and based upon an objective measure such as a neuropsychological mood scale or neuropsychiatric evaluation that complied with the Diagnostic Statistical Manual for Mental Disorders (DSM)-III/IV/V or the International Classification of Diseases (ICD)-7–10.

#### Controls

The comparison groups consisted of participants without depression at the time of study initiation.

#### Outcomes

The dichotomous outcome event of interest was a first-ever stroke during the follow-up period, including fatal and non-fatal ischemic stroke, TIA, and intracerebral hemorrhage (hereinafter referred as “all stroke”).

#### Studies

The study design was a community-, population-, or registry-based longitudinal cohort study reporting relative effect estimates, such as hazard ratios (HRs), relative risk (RRs), and odds ratios (ORs).

### Study Selection

Two reviewers (Shi and Wu) independently evaluated potentially eligible studies that were identified by our search. Articles were screened for eligibility based on a review of the title and abstract only, and disagreements were resolved by consensus. Regarding the remaining papers, their full text was accessed and read independently by the initial two reviewers. When differences of opinion between reviewers occurred, these were resolved by discussion with a third member of the research team, and consensus was thereby reached.

### Data Collection

We developed a specific data extraction sheet. One author (Shi) extracted data from the included studies and another (Wu) used statistical software to check the accuracy of inclusion. Any disagreement was resolved by discussion with the other authors. The data extracted from each study included (1) basic information: author, year of publication, published journals, the number of cases in each group, the proportion of men and women, average age, duration of follow-up time, study design; (2) statistical data (ORs and 95% Cis) for: demographic and social factors, medical history, history of mental disorders, stroke characteristics, impairments, neurocognitive outcome measures, biochemical factors, and other factors. The course of disease was defined as that of acute stage and subacute stage (≤3 months), recovery period (>3 months) (American Psychiatric Association, 2000). When there was any uncertainty about the data, we contacted the corresponding author for clarification. We also collected information suitable for a basic quality evaluation of the studies included, based on the comparability between stroke and non-stroke groups, the risk of selection bias, the evaluation of representativeness of the recruited samples, and the reliability of the depression assessment.

### Statistical Analysis

For studies with data of sufficient quality, and similar in simulation learning and outcome measures, we combined data in a meta-analysis in order to provide a pooled effect estimate. All data were entered into RevMan 5.3^1^, where standardized deviations and 95% CIs were calculated and pooled. The results were expressed as weighted OR with 95% CI for outcomes (Higgins and Green, 2011).

For each analysis, a heterogeneity test was performed using *I*^2^ statistics, which measure the extent of inconsistency among results and is interpreted approximately as the proportion of total variation across studies attributable to heterogeneity and not to chance. *I*^2^ = 25% was considered low, 50% moderate, and 75% high. *I*^2^ values higher than 50% were considered as having substantial heterogeneity, and the random-effects model was therefore applied for analysis of the data. In addition, we performed subgroup analysis according to prespecified variables, including study design and intervention characteristics (i.e., country, follow-up, stroke type). If there had been no statistical heterogeneity, we would have used a fixed-effect model. Subsequently, we performed subgroup analyses according to the study design which was chosen as a potential moderator because different designs were included in the meta-analysis and we considered it is important to be analyzed by subgroup.

To test for publication bias, a funnel plot, which graphs the effect size of each study according to its respective SE, was used. We assumed the existence of publication bias if there were no small studies with effect sizes favoring control groups. A two-tailed *p*-value of less than 0.05 was considered significant (Higgins and Green, 2011).

## Results

### Study Selection

**FIGURE 1:**
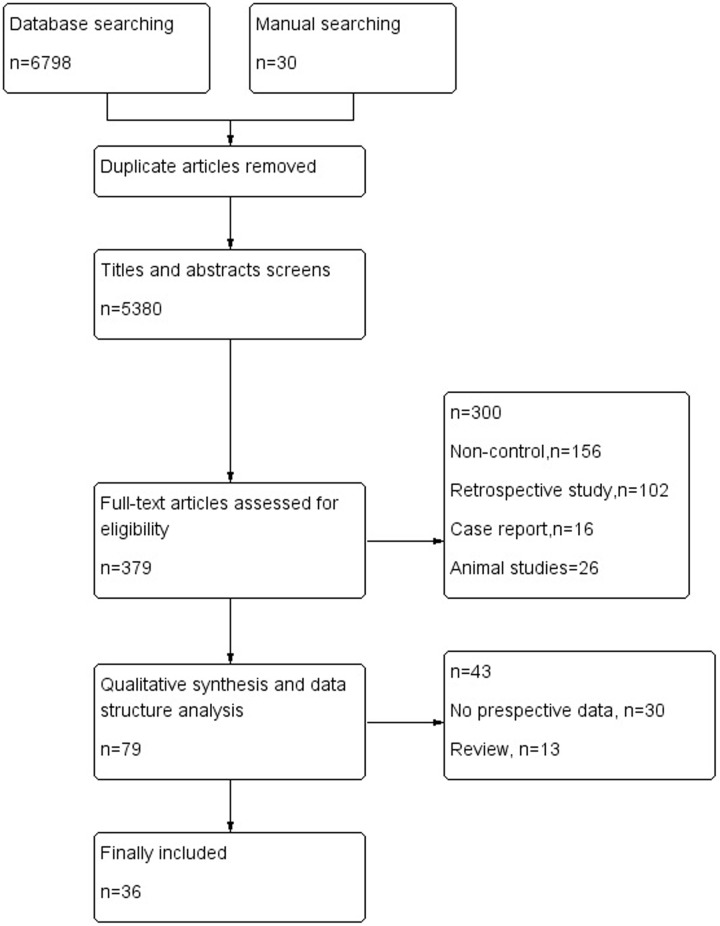
Flow diagram of retrieved, screened, and included studies.

The electronic database search of PubMed, Medline, CENTRAL, EMBASE.com, the Cochrane library and Web of Science provideda total of 6798 citations, and 30 citations were found manually. After removing duplicate manuscripts, 5380 studies remained. Of these, 5000 were excluded based on the title and abstract review, leaving 379 for full text review. These 379 studies with their full text were retrieved and reviewed for eligibility, and 300 were excluded because of ineligible study design and outcome measures. The 79 studies met all the criteria, and these were selected for initial inclusion; after review, a total of 36 articles (Kotila et al., 1998; Pohjasvaara et al., 1998; Aben et al., 2002, 2006; Carota et al., 2005; Tang et al., 2005, 2011; Leentjens et al., 2006; Storor and Byrne, 2006; Brodaty et al., 2007; Lee et al., 2007; Lindén et al., 2007; van de Port et al., 2007; Fuentes et al., 2009; Jimenez et al., 2009; Schepers et al., 2009; Snaphaan et al., 2009; Farner et al., 2010; Ayerbe et al., 2011; Altieri et al., 2012; de Man-van Ginkel et al., 2013; Yang et al., 2013, 2015; Zhang et al., 2013; De Ryck et al., 2014a,b; Li et al., 2014; Ahn et al., 2015; Lewin-Richter et al., 2015; Shi et al., 2015; van Mierlo et al., 2015; Koh et al., 2016; Kootker et al., 2016; Malhotra et al., 2016; Metoki et al., 2016; Tsai et al., 2016) were included in the final analysis (**Figure 1**).

### Study Characteristics

The selected studies included 14791 cases. Follow-up of studies was 2 weeks–15 years. Most of the included studies were combines with the ischemic stroke and hemorrhagic stroke. **Tables 1**, **2** shows the basic characteristics and risk factors for PSD of the included studies.

**Table 1:** Basic characteristics of the included studies.

Reference	Country	Included	Age	Follow-up duration	Stroke assessment	Depression assessment
Shi et al., 2015	China	757	61 @ 11	1 year	MRI or CT	DSM- IV
Lewin-Richter et al., 2015	Germany	96	67 @ 11	6 months	WHO	DSM-IV
Malhotra et al., 2016	Singapore	172		6 months	WHO	CES-D
van Mierlo et al., 2015	Netherlands	344	66.9 @ 12.3	2 years		HADS-D
Yang et al., 2015	China	116	71 @ 8	2 years	MRI	DSM-IV
Li et al., 2014	China	216	68.9 @ 11.3	1 year	CT or MRI	HAM-D
De Ryck et al., 2014a	Belgium	125	69.5 @ 13.0	7 years		CSD
Yang et al., 2013	China	75	66.7 @ 9.3	1 year	CT	DSM-IV
Zhang et al., 2013	China	91		1 year	WHO (CT/MRI)	HAM-D
Lee et al., 2007	China	260	72.0 @ 9.12	1 year	CT/MRI	DSM-IV
de Man-van Ginkel et al., 2013	Netherlands	410	70.0 @ 14.3	2 years	Clinical diagnosis of ischemic infarction	CIDI
De Ryck et al., 2014b	Belgium	222	70.1 @ 13.1	7 years	CT/MRI	CSD
Jimenez et al., 2009	Spain	134	70.4 @ 10.9	6 months	GEECV-SEN	HAM-D
Altieri et al., 2012	Italy	105	64.38 @ 11.2	3 years	MRI/NIHSS	DSM-IV
Fuentes et al., 2009	Spain	185	66.7 @ 10.6	2 years	MRI	DSM-IV
Farner et al., 2010	Norway	126	75.0 @ 11.3	1 year	CT	MADRS
Ayerbe et al., 2011	England	1821		5 years	CT/MRI	HADS
Tang et al., 2011	China	235	67.1 @ 12.1	3 months	CT/MRI	GDS
Schepers et al., 2009	Netherlands	131	57.0 @ 11.1	3 years		CES-D
Snaphaan et al., 2009	Netherlands	420	64.1 @ 12.4	2 months	CT/MRI	HADS
Brodaty et al., 2007	Australia	205	72 @ 9	2 years	CT/MRI	SCID-I
Lindén et al., 2007	Sweden	243		2 years	CT	DSM-III-R
van de Port et al., 2007	Netherlands	165		3 year	CT/MRI	CES-D
Leentjens et al., 2006	Netherlands	165	70 @ 10	1 year	CT	SCID-D
Storor and Byrne, 2006	Australia	100	71.9 @ 13.6	2 weeks	CT	CIDI-DSM-IV
Aben et al., 2006	Netherlands	190	68.5 @ 11.6	1 year	CT/MRI	HAM-D
Carota et al., 2005	Switzerland	273		1 year	CT/MRI	DSM-IV
Tang et al., 2005	China	189	68 @ 11	3 months	CT/MRI	SCID-DSM-IV
Aben et al., 2002	Netherlands	190	68.6 @ 11.7	1 year	CT	HADS
Kotila et al., 1998	Finland	321	70 @ 10	1 year	CT/MRI	BDI
Pohjasvaara et al., 1998	Finland	451	55–85	3 months	MRI	DSM-III-R
Tsai et al., 2016	China	91	64.6 @ 11.0	1 year		DSM-IV
Koh et al., 2016	Korea	52	62.5 @ 15.0	2 years	CT/MRI	GDS
Metoki et al., 2016	Japan	421	73.3 @ 11.1	1 year	MRI	JSS-D
Ahn et al., 2015	Korea	226	68.45 @ 13.00	2 weeks	MRI	K-BDI
Kootker et al., 2016	Netherlands	331	66.7 (24.5–93.5)	2 years	MRI	HADS


**Table 2:** Risk factors for PSD of included studies in the meta-analysis.

Reference	Risk factors associated with PSD
Shi et al., 2015	Y(1, 12, 21); N(2,3,6,10,11,16,17,28)
Lewin-Richter et al., 2015	N(2, 13, 16, 21,22)
Malhotra et al., 2016	Y(2, 10); N(3)
van Mierlo et al., 2015	N(1, 2, 3, 14, 16, 21, 23)
Yang et al., 2015	N(2, 3, 21)
Li et al., 2014	Y(1, 2, 5, 16, 31)
De Ryck et al., 2014a	Y(20); N(1, 2, 3, 4, 6, 10, 16, 17, 21, 23, 24)
Yang et al., 2013	Y(17); N(1, 2, 3, 10, 11, 12, 16,21)
Zhang et al., 2013	Y(1); N(2, 3, 4, 7, 10, 13, 17, 18)
Lee et al., 2007	Y(21, 26); N(1, 6)
de Man-van Ginkel et al., 2013	Y(13), N(9, 10, 23)
De Ryck et al., 2014b	
Jimenez et al., 2009	Y(31)
Altieri et al., 2012	Y(3, 10, 27); N(1, 2, 8, 11, 12, 13, 26)
Fuentes et al., 2009	N(1, 2, 3, 12, 13, 15, 16, 17, 18)
Farner et al., 2010	Y(16, 20); N(17, 21, 23)
Ayerbe et al., 2011	Y(5, 8, 9, 13, 16, 21, 22, 23, 28); N(1, 2)
Tang et al., 2011	Y(1, 3, 9, 10, 16, 18, 21); N(2, 19)
Schepers et al., 2009	Y(1, 25); N(2, 3, 4, 6, 17, 18, 21, 23)
Snaphaan et al., 2009	Y(20, 21, 23); N(1, 2, 3, 5, 10, 13, 17, 18)
Brodaty et al., 2007	Y(9, 10, 20); N(1, 2, 3, 6, 16, 17, 21)
Lindén et al., 2007	Y(2); N(1, 17, 24)
van de Port et al., 2007	Y(8, 18, 20, 23); N(1, 2, 3, 5)
Leentjens et al., 2006	Y(13, 15, 20); N(1, 10, 17, 21)
Storor and Byrne, 2006	Y(13, 14); N(1, 2, 15, 17, 20, 21, 23)
Aben et al., 2006	Y(14, 15, 20); N(1, 2, 13, 17, 18)
Carota et al., 2005	Y(2, 20, 21, 24, 26, 27); N(17)
Tang et al., 2005	Y(1, 3, 9, 17, 18); N(2, 4, 6, 10, 13, 16, 19)
Aben et al., 2002	Y(14, 20); N(1, 2, 13, 17, 21)
Kotila et al., 1998	Y(1, 16, 30); N(2, 5, 10, 17, 18)
Pohjasvaara et al., 1998	Y(13, 16, 18, 20, 23); N(1, 2, 3, 10, 17, 21, 24)
Tsai et al., 2016	Y(1, 13, 16, 23); N(2, 3, 6, 17, 21)
Koh et al., 2016	Y(17); N(2, 13, 18)
Metoki et al., 2016	Y(17)
Ahn et al., 2015	Y(9, 17); N(1, 2, 3, 6, 11, 12, 18, 21)
Kootker et al., 2016	Y(13, 23); N(1, 2, 3, 5, 16, 17, 18, 21)


### Meta-analysis Results

#### Sex

**FIGURE 2:**
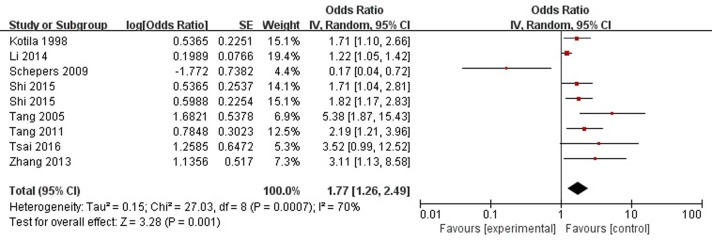
Meta-analysis of sex (female) and PSD risk in the acute stage and subacute stage (≤3 months).

A total of eight articles reported sex (female) was a risk factor for PSD in the acute stage and subacute stage (≤3 months). **Figure 2** shows that there is a high heterogeneity between the trials (χ^2^ = 27.03, *I*^2^= 70%). Therefore the random-effect model was used. Sex (female) was significant associated with PSD [OR = 1.77, 95% CI = 1.26–2.49], but caution should be exercised while drawing conclusions. As a rule of thumb, tests for funnel plot asymmetry should only be used when there are at least 10 studies included in the meta-analysis, so we did not have a test for funnel plot asymmetry in this analysis.

#### Age

**FIGURE 3:**
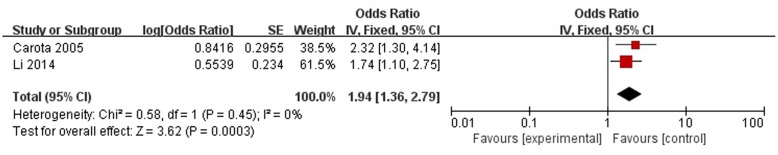
Meta-analysis of age (<70 years) and PSD risk in the acute stage and subacute stage (≤3 months).

A total of two articles reported age (<70 years) was a risk factor for PSD in the acute stage and subacute stage (≤3 months). **Figure 3** shows that there is a low heterogeneity between the trials (χ^2^ = 0.58, *I*^2^= 0%). Therefore the fixed-effect model was used. Age (<70 years) was significant associated with PSD [OR = 1.94, 95% CI = 1.36–2.79], but caution should be exercised while drawing conclusions. As a rule of thumb, tests for funnel plot asymmetry should only be used when there are at least 10 studies included in the meta-analysis, so we did not have a test for funnel plot asymmetry in this analysis.

#### Social Support

**FIGURE 4:**
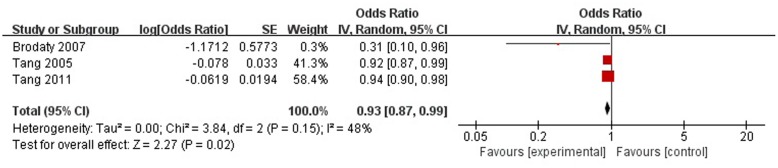
Meta-analysis of social support and PSD risk in the acute stage and subacute stage (≤3 months).

A total of three articles reported social support was a protective factor for PSD in the acute stage and subacute stage (≤3 months). **Figure 4** shows that there is a high heterogeneity between the trials (χ^2^ = 3.84, *I*^2^= 48%). Therefore the random-effect model was used. Social support was associated with PSD [OR = 0.93, 95% CI = 0.87–0.99], but caution should be exercised while drawing conclusions. As a rule of thumb, tests for funnel plot asymmetry should only be used when there are at least 10 studies included in the meta-analysis, so we did not have a test for funnel plot asymmetry in this analysis.

#### History of Mental Illness

**FIGURE 5:**
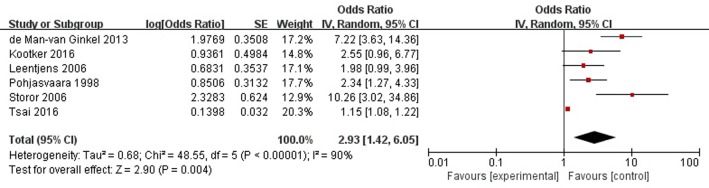
Meta-analysis of history of mental illness and PSD risk in the acute stage and subacute stage (≤3 months).

A total of six articles reported history of mental illness (depression/anxiety/etc.) was a risk factor for PSD in the acute stage and subacute stage (≤3 months). **Figure 5** shows that there is a high heterogeneity between the trials (χ^2^ = 48.55, *I*^2^= 90%). Therefore the random-effect model was used. Psychological history was significant associated with PSD [OR = 2.93, 95% CI = 1.42–6.05], but caution should be exercised while drawing conclusions. As a rule of thumb, tests for funnel plot asymmetry should only be used when there are at least 10 studies included in the meta-analysis, so we did not have a test for funnel plot asymmetry in this analysis.

#### Neuroticism

**FIGURE 6:**
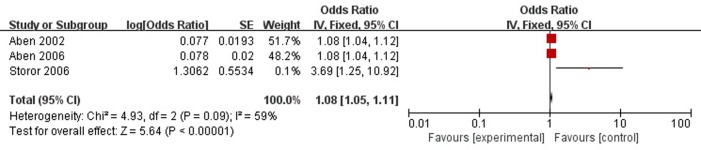
Meta-analysis of neuroticism and PSD risk in the acute stage and subacute stage (≤3 months).

A total of three articles reported neuroticism was a risk factor for PSD in the acute stage and subacute stage (≤3 months). **Figure 6** shows that there is a high heterogeneity between the trials (χ^2^ = 4.93, *I*^2^= 59%). Therefore the random-effect model was used. Neuroticism was significant associated with PSD [OR = 1.08, 95% CI = 1.03–1.14], but caution should be exercised while drawing conclusions. As a rule of thumb, tests for funnel plot asymmetry should only be used when there are at least 10 studies included in the meta-analysis, so we did not have a test for funnel plot asymmetry in this analysis.

#### Family History of Mental Illness

**FIGURE 7:**
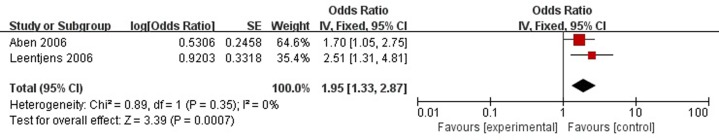
Meta-analysis of family history and PSD risk in the acute stage and subacute stage (≤3 months).

A total of two articles reported family history of mental illness was a risk factor for PSD in the acute stage and subacute stage (≤3 months). **Figure 7** shows that there is a low heterogeneity between the trials (χ^2^ = 0.89, *I*^2^= 0%). Therefore the fixed-effect model was used. Family history was significant associated with PSD [OR = 1.95, 95% CI = 1.33–2.87], but caution should be exercised while drawing conclusions. As a rule of thumb, tests for funnel plot asymmetry should only be used when there are at least 10 studies included in the meta-analysis, so we did not have a test for funnel plot asymmetry in this analysis.

#### Severity of Stroke

**FIGURE 8:**
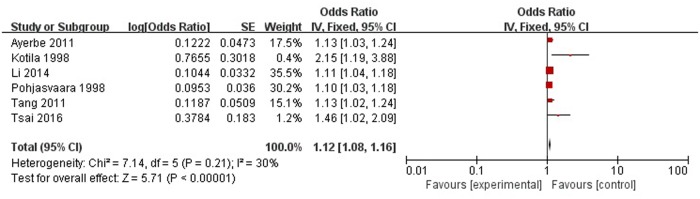
Meta-analysis of severity of stroke and PSD risk in the acute stage and subacute stage (≤3 months).

A total of six articles reported severity of stroke was a risk factor for PSD in the acute stage and subacute stage (≤3 months). **Figure 8** shows that there is a low heterogeneity between the trials (χ^2^ = 7.14, *I*^2^= 30%). Therefore the fixed-effect model was used. Severity of stroke was significant associated with PSD [OR = 1.12, 95% CI = 1.08–1.16], but caution should be exercised while drawing conclusions. As a rule of thumb, tests for funnel plot asymmetry should only be used when there are at least 10 studies included in the meta-analysis, so we did not have a test for funnel plot asymmetry in this analysis.

#### Level of Handicap

**FIGURE 9:**
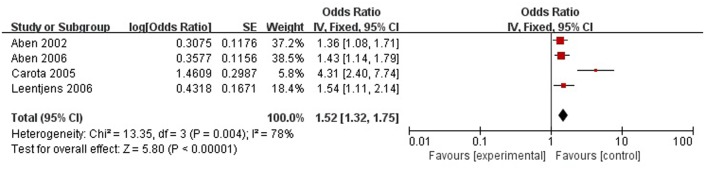
Meta-analysis of level of handicap and PSD risk in the acute stage and subacute stage (≤3 months).

A total of four articles reported level of handicap was a risk factor for PSD in the acute stage and subacute stage (≤3 months). **Figure 9** shows that there is a high heterogeneity between the trials (χ^2^ = 13.35, *I*^2^= 78%). Therefore the random-effect model was used. Level of handicap was significant associated with PSD [OR = 1.52, 95% CI = 1.32–1.75] in acute stage and subacute stage (≤3 months), but caution should be exercised while drawing conclusions. As a rule of thumb, tests for funnel plot asymmetry should only be used when there are at least 10 studies included in the meta-analysis, so we did not have a test for funnel plot asymmetry in this analysis.

**FIGURE 10:**
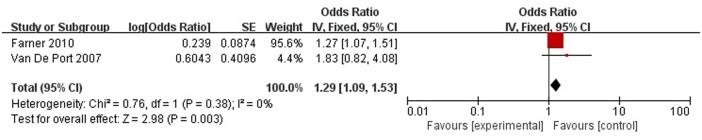
Meta-analysis of level of handicap and PSD risk in the recovery period.

A total of two articles reported level of handicap was a risk factor for PSD in the recovery period (>3 months). **Figure 10** shows that there is a low heterogeneity between the trials (χ^2^ = 0.76, *I*^2^= 0%). Therefore the fixed-effect model was used. Level of handicap was significant associated with PSD [OR = 1.29, 95% CI = 1.09–1.53] in recovery period, but caution should be exercised while drawing conclusions. As a rule of thumb, tests for funnel plot asymmetry should only be used when there are at least 10 studies included in the meta-analysis, so we did not have a test for funnel plot asymmetry in this analysis.

#### Level of Independence

**FIGURE 11:**
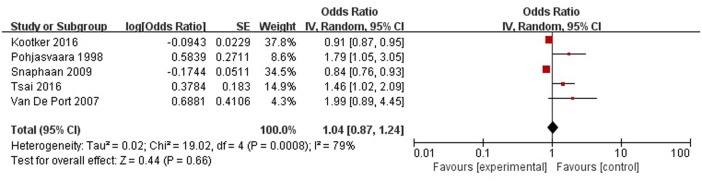
Meta-analysis of level of independence and PSD risk in the acute stage and subacute stage (≤3 months).

A total of five articles reported level of independence was a risk factor for PSD in the acute stage and subacute stage (≤3 months). **Figure 11** shows that there is a high heterogeneity between the trials (χ^2^ = 19.02, *I*^2^= 79%). Therefore the random-effect model was used. Psychological history was significant associated with PSD [OR = 1.04, 95% CI = 0.87–1.24], but caution should be exercised while drawing conclusions. As a rule of thumb, tests for funnel plot asymmetry should only be used when there are at least 10 studies included in the meta-analysis, so we did not have a test for funnel plot asymmetry in this analysis.

### Other Data

Due to the limitations of some included studies, some data sets were insufficient to complete the meta-analysis (*n* < 2) and, in other studies, the original data could not be obtained. We show the unanalyzed data here, as follows:

#### Education

A total of four studies reported that education (>8 years) was a protective factor in the acute and subacute stages (≤3 months).

#### Predisposing Illness

Most of the included studies reported that predisposing illness, such as hypertension, diabetes mellitus, hyperlipidemia, atrial fibrillation, and myocardial infarction, was not associated with PSD.

#### Location

In the included studies, the left hemisphere had an association with PSD in the acute and subacute stages. Brain damage in the left frontal lobe and left basal ganglia was associated with PSD.

#### Biochemical Factors

Most biochemical factors, such as interleukin-1β (IL-1β) and intercellular cell adhesion molecule-1 (ICAM-1) were not associated with PSD, but the levels of brain-derived neurotrophic factor (BDNF) and leptin were risk factors for PSD.

## Discussion

Post-stroke depression is considered the most frequent and serious neuropsychiatric consequence of stroke (Cully et al., 2005). The reported prevalence of PSD varies widely, ranging from 25 to 79% (Gillen et al., 2001). Patients with PSD have more functional disability, poorer rehabilitation outcomes, and increased morbidity and mortality in the first year after stroke onset (Williams et al., 2004). Therefore, it is very important to find out the risk factors for PSD.

Views regarding the risk factors for PSD divide into two opposites: some propose a primary biological mechanism interpreting PSD (Li et al., 2014), whereas others claim that PSD is caused by social and psychological stressors owing to stroke (Farner et al., 2010). Besides some studies have come to different conclusions, for example, Tang et al. (2011) suggested that gender (female) was the important factor regarding PSD, while Ayerbe et al. (2011) failed to find this association. These controversies and other similar divergences all point toward the need for meta-analysis of this important topic.

This meta-analysis was conducted with 14,791 patients suffering from PSD. Many risk factors have been investigated over the last three decades; controversy exists concerning risk factors for the development of PSD. This meta-analysis has revealed that a history of mental disorders, stroke severity, gender and age are significantly associated with PSD.

### History of Mental Disorders

Our meta-analysis showed that, in the acute and subacute stages (≤3 months), psychiatric history (i.e., depression, anxiety) was associated with PSD [OR = 3.95, 95% CI = 1.88–8.32], which suggests that a medical history of depression or other psychiatric disorders is one of the leading risk factors for PSD. Another study confirmed previous depressive episodes to be a predictor for PSD (Paolucci et al., 2005). Paolucci et al. (2006) found that, in the recovery period (>3 months), a psychiatric history was also associated with PSD (*p* < 0.001, χ^2^= 40.14) (Paolucci et al., 2006). In psychiatry, a mental illness may recur many times, and can lead to other mental illnesses (Burns et al., 2007). Accordingly, a history of psychiatric disorders could be a predisposing factor for PSD, simultaneously as a good indicator for PSD prevention.

Other major factors contributing to PSD are neuroticism and a family history of mental disorders. Our results suggest that neuroticism is strongly associated with PSD in the acute and subacute stages; this suggestion is supported by a study (Liu et al., 2015) indicating that high neuroticism conferred a greater risk of PSD (Andersen et al., 1995). Therefore, a personality assessment, as part of a screening test, could be useful for those at risk of developing depression. Moreover, both Aben et al. (2006) and Leentjens et al. (2006) showed that a family history of mental illness was associated with PSD. Mental illnesses are complex disorders resulting from the combined action of genetic and environmental factor. Thus, PSD may be explained by a genetic inheritance of family mental illness.

### Stroke Characteristics

Brain damage caused by stroke is located mostly in the frontal lobe and basal ganglia where are highly correlated with emotional processing (Hornak et al., 1996; Pell and Leonard, 2003). Our meta-analysis revealed that stroke severity could be one of the most important risk factors for PSD, which conformed to the hypothesis that there may be an association between the extent of brain damage and depression (Vataja et al., 2004). Movement disorders, dysfunction, and life obstacles caused by brain damage could probably decline the self-confidence of patients and sudden stroke can also be regarded as a negative event for the sufferers, which might increase the incidence of depression. Consequently, stroke severity was identified as a vital factor for PSD because of its influence on the levels of handicap and independence that should remain as significant risk factors considered into the prevention of PSD.

There is a long-standing debate over the association between lesion locations and PSD. Robinson et al. (1984) and Starkstein and Robinson (1989) found that patients with left hemispheric lesions are more depressive than those with right hemispheric lesions and the severity of PSD is closely related to the extent of frontal lobe damage. But some other researches (Gainotti et al., 1999; Carson et al., 2000; Gainotti and Marra, 2002; Kutlubaev and Hackett, 2014; Wei et al., 2015) rejected this view, they suggested that lesion locations are not associated with PSD, and that the psychological model would play a role in the risk of PSD. In our study, we found that the damage of left hemisphere, especially in the left frontal lobe and left basal ganglia, had a close association with the extent of PSD in the acute and subacute stages, which suggested that the location of lesions do correlate with PSD. Our results are also supported by Vataja et al. (2001) and Rajashekaran et al. (2013). The possible reasons for this conjecture were that the left hemisphere is the dominant hemisphere responsible for positive emotions and language, and that the degree of neurological deficits in the left hemisphere is more serious in stroke patients according to the contrast of imaging data (Davidson and Irwin, 1999).

The frontal lobe and basal ganglia charge the heart of the emotional network (Jastorff et al., 2015), and these brain areas are more likely to change and then lead to depressive symptoms. At the same time, it is worth noting that using repetitive transcranial magnetic stimulation to stimulate focal brain is found more effective when it is administered to the left dorsolateral prefrontal cortex in patients with depression (Robinson and Jorge, 2016). This phenomenon also suggests that the prefrontal cortex plays an important role in depression. Of course, we cannot ignore the other phenomenon that in clinical observation, there are many patients with occipital lobe stroke or parietal lobe stroke having depressive symptoms but without obvious lesion locations in the emotional network. Accordingly, we should pay attention to the important role of psychological factors in the development of PSD (Gainotti et al., 1999, 2001). As we all know, stroke, as a serious stress event, is a heavy psychological blow to stroke patients. For instance, the neurologic impairment will cause a decline in or loss of the ability to work, which would make the stroke patients inevitably suffer from enormous social psychological pressure and finally lead to despair (Vallury et al., 2015; Eriksen et al., 2016). Therefore, psychological model is also play a role in the risk of PSD. Apart from stroke, serious diseases such as chronic heart disease and spinal cord injury, can also cause great physical and mental trauma leading to serious problems and a heavy family burden. However, there were statistics have shown that the incidence of depressive symptoms of the above diseases was lower than that of stroke (Fedoroff et al., 1991), which suggests that the psychological model just plays a part in the onset of PSD. Above all, we believe that stroke accompanying with the brain damage in the areas of the dominant emotional hemisphere or the emotional circuit, is more likely to occur depressive symptoms; and psychological factors also play an important role in the development of PSD. The differences found in previous findings are closely related to the design of this study. The different definitions of stroke, the difference in the imaging equipment, and the high heterogeneity of the subjects comprehensively lead to different results. A study with a large sample size, strict design, and unified diagnostic standards should be able to solve the above contradictions.

### Personal and Social Factors

Our meta-analysis found that female gender was a significant risk factor for PSD in the acute and subacute stages, which was supported by previous studies. Paolucci et al. (2006) and Schepers et al. (2009) similarly suggested that gender was associated with PSD in the recovery period. Some studies considered that hormonal changes perhaps play a role in the occurrence of PSD because of their impacts on the moods of female patients (Beekman et al., 1999; Katona and Livingston, 2000). Stroke is a serious disease and a heavy blow to the patients as well. Women’s coping methods dealing with this situation are relatively inadequate and their psychological quality is also poor (Stewart et al., 2001; Berg et al., 2003; Verdelho et al., 2004). These help to explain why older women, especially those living alone, are more likely to get PSD. Moreover, possibly due to the decreased mental capacity and the slower recovery those reducing the quality of life and increasing mental pressure, the occurrence of depression may be more common in the middle-aged stroke patients than that in the elderly ones (>70 years), which has been shown in the included studies. It seems to be more difficult for the middle-aged (<70 years) to face their physical disorders and work capacity loss caused by the disease.

In the early stage of stroke, sudden behavioral disorders lead to a reduction in the number of patients engaging in social interactions (Boden-Albala et al., 2005). As social isolation has a negative impact on general health, it might play a role in the association with PSD (Tomaka et al., 2006). Limited activity gradually affects the mood and self-confidence of the patient; in their times of difficulty and stress, strong family and social support is extremely helpful and of importance for them, as it may contribute to improve their motivation adjusting to the disability and arouse their enthusiasm participating in social activities (Tsouna-Hadjis et al., 2000; Ayerbe et al., 2013; Northcott, 2013).

Patients with long-term education may develop better self-adjustment abilities (Ellen and Stéphan, 2013), which play a role in accommodating the change of PSD. Our meta-analysis revealed that living conditions at the time of stroke (alone/with family) were not associated with depression, so were housing conditions. In some studies (Robinson and Price, 1982), living alone did not predict depression, but in one study (Astrom et al., 1993) an association was found.

### Other Factors

Our meta-analysis showed that the level of handicap was associated with PSD both in the acute and subacute stages and the recovery period. Burvill et al. (1997) and De Ryck et al. (2014a) and also suggested that the level of handicap was a risk factor for PSD in the recovery period. The level of handicap reflects the degree of disability, as well as the degree of brain damage. A high level of handicap would seriously impact the patients on their life and work, leading to great physiological and psychological trauma, and eventually results in PSD. Therefore, the level of handicap could also be a good indicator for PSD prevention.

It cannot be ignored that secondary neurodegeneration has a serious impact on PSD (Loubinoux et al., 2012). In secondary neurodegeneration, nerve damage after focal cerebral infarction not only affect the local lesions, but also affect the nerve fibers in remote brain areas (Liu et al., 2012). The axons connecting with the infarct neurons will have Wallerian degeneration and cause neuronal loss in remote brain areas (Dihne et al., 2002). Robinson and Bloom (1977) suggested that dysfunction of the (cortico)-striato-pallido-thalamic-cortical circuit would lead to depressive symptoms. Due to the influence of secondary neurodegeneration, even the stroke lesion is not included in the emotional circuit; this circuit could also be affected by the remote cerebral area. Though the thalamus and substantia nigra do not belong to the blood supply area of the middle cerebral artery (MCA), studies (Nakane et al., 1997; Zhao et al., 2001) have shown that MCA occlusion (MCAO) still affects excitability in the brain areas of the thalamus and substantia nigra, resulting in mood disorders. In addition, secondary neurodegeneration can also cause the release of inflammatory factors (Block et al., 2005) and aggravate the inflammatory response of remote cerebral area, which would play a role in the risk of PSD.

Robinson et al. (1986) proposed a theory about primary biological mechanism of PSD, following that more and more researchers focused on the biological principles of PSD. Li et al. suggested that inflammatory factors such as interleukin 1 (IL-1), interleukin 6 (IL-6), and interferon gamma (IFNγ), were important factors responsible for PSD (Li et al., 2014). In addition, other inflammatory factors, such as tumor necrosis factor alpha (TNF-α) (Spalletta et al., 2006), interleukin-8 (IL-8) (Spalletta et al., 2006), interleukin-18 (IL-18) (Kang et al., 2016), and high-sensitivity C-reactive protein (Hs-CRP) (Cheng et al., 2017) have also been found to play an important role in the onset of PSD. Under the stress of stroke, inflammatory factors increase dramatically. There are several plausible mechanisms for this association. First, IL-18 could induce the expression of indoleamine 2,3-dioxygenase (IDO), and overexpression of IDO can cause the depletion of 5-hydroxytryptamine (5-HT), leading to depressive symptoms (Maes et al., 2002; Spalletta et al., 2006). Second, Hs-CRP could increase the hyperintensity burden of white matter, which leads to depressive symptoms (Schmidt et al., 2011). At the same time, Cheng et al. (2017) suggested that homocysteine (HCY) also play a role in the risk of PSD, and overexpression HCY produced by stroke could affect the expression of BDNF (Folstein et al., 2007; Obeid et al., 2007). As an important nutrient for neurons, decreased release of BDNF can cause hippocampal atrophy and reduce synaptic plasticity (Failla et al., 2016), leading to the occurrence of depression. At present, the neurobiological mechanisms of PSD remain unclear, but the inflammatory factors and other biochemical factors [i.e., BDNF (Chen et al., 2005) and leptin (Söderberg et al., 1999)], provide new directions for the prediction of PSD.

## Limitations

Although there were 36 studies included in our analysis, most primary data from these examined studies were not available, thus very little data could be effectively used in our analysis. For the same reason, we could not carry out subgroup analysis. Furthermore, the reliability of the selected studies was reduced due to the high heterogeneity of the data while most of the meta-analyses concerned the acute and subacute stages. Consequently, the data reflecting the outcomes in the recovery period were insufficient. In the included literature, there were some differences in the experimental designs and most studies included both ischemic stroke and hemorrhagic stroke, which could have affected the quality of the meta-analysis. To prove the significant advantages, large-scale, multiple-term, and high-quality studies are necessary.

## Conclusion

There are many factors that have effects on PSD. The severity of stroke is an important factor in the occurrence of PSD. Mental history is a possible predictor of PSD. Prevention of PSD requires social and family participation.

## Author Contributions

The literature was screened and methodological quality was assessed independently by YS and DY. WW provided overall expertise on post-stroke depression. YS and YZ performed the meta-analysis. YS drafted the manuscript. All authors approved the final version and agree to be accountable for this work.

## Footnotes

1 http://tech.cochrane.org/revman/download

## Abbreviations

A (personal and social factors): 1(sex), 2(age), 3(education), 4(employment), 5(alone), 6(marital status), 7(housing conditions), 8(social activities), 9(lack of family and social support)
B (medical history): 10(predisposing illness), 11(alcohol abuse), 12(cigarette smoking)
C (history of mental disorders): 13(mental disease), 14(neuroticism), 15(family history of disease)
D (stroke characteristics): 16(severity), 17(location), 18(type)
E (impairments): 19(physical condition before stroke), 20(level of handicap), 21(cognition), 22(Inability to work), 23(level of independence)
F (neurocognitive outcome measures): 24(aphasia/dysphasia), 25(neglect)
G (others): 26(overt sadness), 27(crying), 28(use of antidepressive at baseline), 29 (length of hospital stay), 30(active rehab program), 31(biochemical factors). Y, significant risk factors N, no significant risk factors.
